# Mobile App–Based Intervention for Adolescents With First-Episode Psychosis: Study Protocol for a Pilot Randomized Controlled Trial

**DOI:** 10.3389/fpsyt.2019.00027

**Published:** 2019-02-05

**Authors:** Sara Barbeito, Teresa Sánchez-Gutiérrez, María Mayoral, Miguel Moreno, Sergio Ríos-Aguilar, Celso Arango, Ana Calvo

**Affiliations:** ^1^Faculty of Health Sciences, Universidad Internacional de la Rioja (UNIR), Logroño, Spain; ^2^Department of Child and Adolescent Psychiatry, Hospital General Universitario Gregorio Marañón School of Medicine, Universidad Complutense, Instituto de Investigación Sanitaria Gregorio Marañón (IiSGM), CIBERSAM, Madrid, Spain; ^3^Mental Health Network of Guipuzcoa, Biodonostia, San Sebastian, Spain; ^4^School of Engineering and Technology, Universidad Internacional de La Rioja (UNIR), Logroño, Spain

**Keywords:** early onset psychosis, mobile treatment, psychotherapy, adolescents, online treatment

## Abstract

**Introduction:** Previous studies have shown an improvement in the access to treatment for patients with first-episode psychosis (FEP), specifically young patients, through mobile app–based interventions. The aim of this study is to test the effectiveness of a mobile app–based intervention to improve community functioning in adolescents with FEP. Mobile app–based interventions could increase quality of life and disease awareness, which improve adherence to treatment and reduce the frequency of relapses and rehospitalizations in adolescents with FEP.

**Methods:** This article describes a mobile app treatment and the pilot trial protocol for patients with FEP. We will perform a single-blind randomized clinical trial (RCT) including patients with FEP aged 14–19 years recruited from Gregorio Marañón Hospital, Madrid, Spain. Patients will be randomly assigned to an intervention group, which will receive treatment as usual plus five modules of a psychological intervention through the mobile app (psychoeducation, recognition of symptoms and prevention of relapses, problem solving, mindfulness, and contact wall), or to a control group (standard care). The effectiveness of the intervention will be assessed by means of an extensive battery of clinical tests at baseline and at 3 months of follow-up. The primary outcome is reduction in psychotic and depressive symptoms; secondary outcomes comprise adherence, awareness, use of drugs, and quality of life. Data will be analyzed on an intention-to-treat (ITT) basis. Mixed model repeated-measures analysis will be used to explore the following effect: group × time interaction between the control group and the intervention group for clinical and functional variables during the follow-up period.

**Discussion:** This is an innovative study for the assessment of a psychological intervention through a mobile app for patients with FEP during the critical period. This pilot RCT is intended to be a precursor to larger studies, which in turn could facilitate dissemination of mobile app therapy for patients with FEP.

**Ethics and Dissemination:** The local ethics committee approved the study protocol. All participants must sign the informed consent, to participate. After finalizing the study, the results will be published.

**Trial registration:** NCT03161249. NCT clinicaltrials.gov. Date of registration in primary registry 02 May 2017. clinicaltrials.gov.

## Introduction

Psychotic disorders typically emerge in adolescence and young adulthood. While some patients recover fully, many experience persistent difficulties, or remain vulnerable to future episodes. The course of psychotic disorders with onset in childhood or adolescence is variable, although overall prognosis is usually worse than for adults (1, 2). Moreover, adolescence is a critical period for biological, psychological, and social development. Consequently, the emergence of a severe psychiatric disorder at this stage could have a strong impact on personal achievements and cause major disability (3). Five years after the onset of first-episode psychosis (FEP), the relapse rate reaches 70–80% (4); therefore, early intervention is a priority. Psychological intervention may significantly improve symptomatology, functional recovery, prognosis, and quality of life in FEP patients (5). Available approaches usually take the form of assertive outreach programs (e.g., cognitive behavior therapy, medication, family support), which include a combination of psychoeducational interventions aimed at family groups (6–8). However, some studies show that the benefits of these kinds of interventions are not maintained in the long term (9–12). In this context, online interventions could be a therapeutic complement to maintain the effectiveness of psychosocial interventions that have already shown their effectiveness in the short and medium term (13).

With the rise of new technologies, research on psychological interventions during adolescence has incorporated the use of various electronic applications, social networks, and other similar tools to provide new methods/routes of communication between therapists and patients. This new therapeutic approach may provide patients with personalized, flexible, and evidence-based interventions in their communities and even in their own homes (14).

Although the use of technological advances in psychiatric treatment is relatively recent, the availability and sophistication of technologies are growing. This is particularly true for smartphones and mobile applications, which are one of the most rapidly expanding and adopted forms of technology in history (15). Available research suggests that in western countries, up to 90% of people diagnosed with FEP have access to a smartphone (16, 17).

Some studies have reported results for smartphone-based interventions and conclude they are feasible, attractive, and safe in young patients with FEP or psychosis (18–20), although it must be stressed that adherence depends on these factors (19). Thus, most patients perceive this type of intervention to be positive and useful and show adequate adherence to the intervention during follow-up (21). With respect to specific diagnoses, a recent study shows the feasibility, acceptability, and preliminary efficacy of such interventions for patients with schizophrenia (22). In terms of symptoms, studies report improvements in affective and psychotic symptoms, number of admissions, sociability, and adherence to treatment (20, 21, 23). Nevertheless, despite the fact that some studies report that these on-line interventions are feasible and safe in young patients, more randomized controlled clinical trials are needed to consistently assess the effectiveness of digital interventions in FEP (24).

The aim of this study is to test the effectiveness of a mobile app–based intervention to improve community functioning in adolescents with FEP. It may also increase quality of life and disease awareness, which will lead to greater adherence to treatment and fewer relapses and rehospitalizations.

To our knowledge, this is the first clinical study to evaluate the effectiveness of an online intervention through a mobile app specifically designed for adolescents with FEP, as a complement to their usual treatment. We hypothesized that participants in the app group would find the online intervention acceptable, useable, attractive, and helpful. We also hypothesized that participants' in the app group would experience fewer psychotic and affective symptoms and that global functioning and adherence to treatment and quality of life would be better than in the treatment as usual group. We provide a detailed description of the study design, patient selection and evaluation, and the psychological treatment modules under study (implemented by a mobile app).

## Aims

The aim of this study is to evaluate the efficacy of an online intervention focused on symptoms (psychotics and depressive) compared to a treatment as usual in adolescent patients with early psychosis on several variables including adherence, awareness, prognosis, use of drugs, and quality of life.

## Methods and Analysis

### Design

We designed a pilot randomized controlled trial (RCT) of a smartphone application used in conjunction with usual care and compared it with usual care alone in adolescents with FEP. The trial is to be performed single-blind and at a single center.

### Patients' Recruitment

We will recruit patients from the Child and Adolescent Psychiatry Department of Hospital General Universitario Gregorio Marañón in Madrid, Spain between 2018 and 2019. Recruitment will begin after the protocol has been published. Patients who agree to participate in the study will be assessed and randomly assigned to the intervention group (mobile app intervention) or to the control group (standard care). The intervention group will receive treatment as usual plus a psychological intervention through a mobile app. The control group will receive only the treatment as usual (pharmacotherapy together with regular sessions with a psychiatrist and/or clinical psychologist). The pharmacological treatment will be that prescribed by the patient's psychiatrist.

#### Patients' Inclusion Criteria

The inclusion criterion for patients will be age 14–19 years with the presence of at least one positive psychotic symptom (delusions or hallucinations) before age 19 years and 1 of the following diagnoses from DSM-5 (25): schizophrenia, schizoaffective disorder, schizophreniform disorder, bipolar disorder, major depressive disorder with psychotic features, brief psychotic disorder, or psychosis not otherwise specified. They must also be fluent in Spanish and give their written informed consent to participate. In the case of minors, parents or legal guardians will be required to provide their written informed consent prior to inclusion. The patient must also agree to participate. Furthermore, all patients will have to have spent at least 2 months free from hallucinatory or delusional psychotic symptoms.

#### Patients' Exclusion Criteria

*Exclusion criteria*: Patients with intellectual disability, organic brain disorders, comorbid conditions that could hinder communication, or drug abuse as a primary diagnosis will be excluded.

### Randomization and Blinding

Patients will be randomized to the groups (1:1) using the program “random allocation software.” The person in charge of the assessments will not be involved in the treatment program and will be blind to this process. Study evaluators will be blind to the treatment branch and trained not to comment on it. In emergency cases, the patient will be excluded from the study. This situation will be indicated in the protocol, although the patient will continue with his/her usual treatment and be treated by his/her reference psychiatrist, who will be informed of this situation and ensure that appropriate clinical treatment is administered.

**Figure 1:**
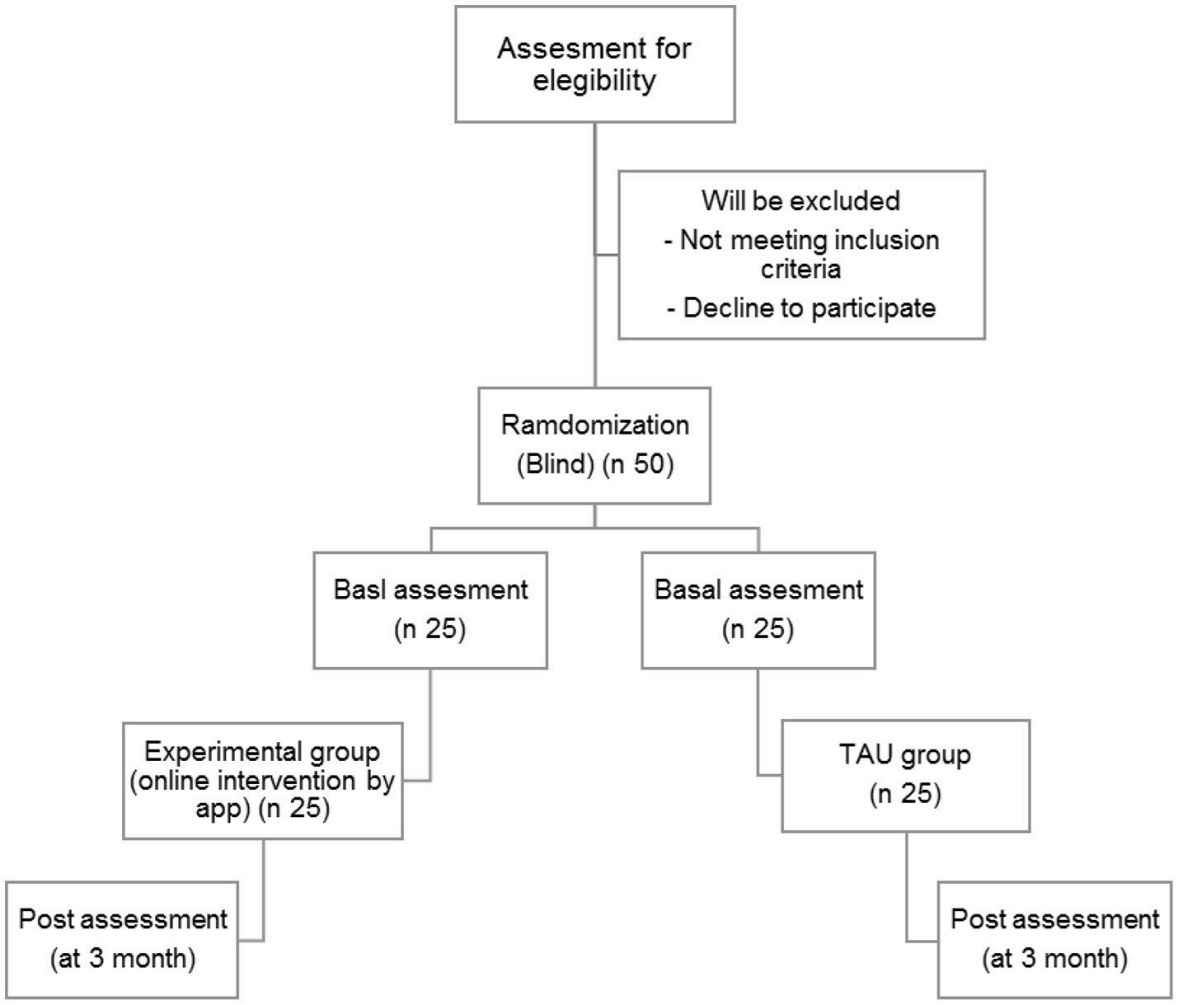
Participant flow diagram.

The flow diagram of the study can be seen in Figure 1.

### Ethical Criteria and Ethics Committee

The project will be conducted according to local regulations and the internationally established principles of the Declaration of Helsinki (Fortaleza, Brazil, 2013). The study and the protocol were approved by the Clinical Research Ethics Committee of each participating health center (UNIR University and Gregorio Marañón Hospital: Certificate no. 06/2017). Written informed consent will be required for all participants, in the case of minors, parents or legal guardians will be required added to provide their written informed consent prior to inclusion.

### Sample Size Calculation

Few previous longitudinal studies have analyzed children and adolescents with the characteristics of the patients in the present study (26, 27). Sample size was calculated based on data published in the literature related to the main issue addressed in the study (28), namely, mu1 (42.5) and mu2 (31.9), sigma value (11.54), value for α (0.05), and value for desired power (0.80). The proposed sample size was 50 patients-−25 per group—which is anticipated to be further reduced by loss to follow-up, resulting in an approximate final sample of 38–42 patients. In order to obtain a power of 80% to detect differences from the null hypothesis, H0: μ1 = μ2, using inferences to compare the means of two independent samples, with a significance level of 5%, we need to include at least 19 patients in the experimental group and 19 patients in the control group. Recruitment of participants is adjusted to 25 per group to account for possible dropouts.

### Clinical Assessment Criteria

Data on the major clinical and demographic variables will be entered on a data collection form. All patients will be assessed by blind raters at baseline and at 3 months after inclusion in the study, after signature of informed consent, and randomization. During follow-up, patients who use the app will be “monitored” by mental health professionals. Their reference clinicians will fully informed, and families will know that the adolescents are participating in the study. Any emergency or major side effect in either of the study arms will be pointed out, and the patient will be removed from the study. If necessary, the patient will be referred to the emergency department.

All the instruments used in the study have appropriate psychometric properties (29–31).

#### Demographic Data

Age, sex, level of education, living arrangements, and employment or educational status will be collected from each participant. All diagnoses will be made by the patient's reference psychiatrist, and assessments will be conducted blind by psychiatrists/psychologists experienced in child and adolescent psychiatric disorders.

#### Evaluation of Patients' Symptoms

Psychotic symptoms will be assessed using the Positive and Negative Syndrome Scale PANSS (32), which is composed of 30 items covering positive and negative symptoms and the general psychopathology of the disorder. Each item is measured using a 7-point Likert scale reflecting the severity of symptoms from 1 (none) to 7 (extreme symptoms). The scale has been validated for the Spanish population (33). In addition, Positive symptoms will be assessed using SAPS (34) and negative symptoms using SANS (35), both of which were validated in Spanish by Vazquez in 1989 (36, 37). SAPS has 34 items and SANS 25. Both have good psychometric properties.

Trait and state anxiety will be measured using the State-Trait Anxiety Inventory (STAI) (38). This instrument has two parts: one that assesses the anxiety experienced by patients during the previous week (namely “state”) using 20 items with 4 response options; and another that assesses their usual reactions to specific situations (namely “trait anxiety”), also using 20 items with 4 response options. The STAI was adapted from the Spanish study in 1978 by Bermudez (39), who found that in these items, the mean and the reliability (Cronbach alpha and test-retest correlation) were similar to those of the original version (40).

Mood symptoms will be evaluated using the Hamilton Rating Scale for Depression (HRSD) (41) validated for the Spanish population (42). This instrument is in three different versions (5, 7, 21 items) and has good psychometric properties. The 21-item version will be used for this study.

#### Evaluation of Patients' Awareness of Their Disease

We will measure the patient's insight using the Scale to Assess Unawareness of Mental Disorders (SUMD) (43), which provides a broad assessment of the patients' thoughts and beliefs regarding their disease and medication. The Spanish adaptation of the SUMD is equivalent to the original, with similar reliability and external validity to those of the original version (44). The 9-item version will be used for this study.

#### Assessment of Prognosis

We will use the Strauss and Carpenter Prognostic Scale (45–47), an instrument that assesses the best status of the patient in the previous year in four areas: hospitalization, work, social activity, and global functioning. The scale is an interview-administered instrument with Likert-type response options ranging from 0 to 4; higher scores indicate a better prognosis. The scale was validated for schizophrenia studies in the Spanish population (48).

We will also assess the overall functioning of patients using the Spanish version of the GAF, which is recommended for Axis V disorders in the multiaxial system of DSM-IV-TR (49) and is based on the opinion of clinicians regarding the level of general activity and functioning of patients (severity). The scale was validated in patients with severe mental disorders (50).

In addition, the level of functioning will also be measured using the Children's Global Assessment Scale (C-GAS) (51, 52). GAF or C-GAS will be selected according to the patient's age.

#### Assessment of Adherence to Treatment

We will measure this variable using the Morisky Medication Adherence Scale (53), which has been validated for the Spanish population (54). It has four questions and assesses the attitudes of the patients toward their treatment. Patients with a score of four will be considered as having “good” adherence, while those with a score between 0 and three will be classified as having “poor” adherence.

#### Assessment of Drug Use

The use of drugs will be determined using the Addition Severity Index (55), which was adapted for the Spanish population by Guerra in 1992 (56) and evaluates substance abuse problems. It is a semi-structured interview with 142 items. Data correspond to the last 30 days in both objective and subjective reports.

#### Assessment of Quality of Life

Quality of life will be assessed using the World Health Organization Quality of Life WHOQOL-BREF Questionnaire (57). The short version is composed of 26 items exploring 4 dimensions (physical health, psychological health, social relationships, and the environment). Spanish trials were the first to report on the validity of the WHOQOL in people with schizophrenia (58).

Quality of life will also be assessed using the EuroQoL questionnaire (59), which measures five dimensions (mobility, self-care, usual activities, pain/discomfort, and anxiety/depression). It also contains a visual analog scale represented by a vertical line on which subjects rate their self-perceived health status from 0 (the worst) to 100 (the best imaginable health status). The validated Spanish version of this scale is simple and practical (60).

### Data Management

Data confidentiality will be ensured using a coded database. In order to further guarantee confidentiality, access will be restricted to the therapist and the investigators. All data will be anonymous and confidential and will have a code. All the documents recorded to collect data will be saved and filed following the regulations in force in Spain (*General Regulation on Data Protection in force in 2018*).

### Software Development

We will develop an Android Mobile app with a web support and administration application and a cloud-based backend database, which will register fine-grain interactions between the users and the app in order to generate relevant granular data and key performance indicators and thus support a wide range of further analyses.

The application will be designed and developed using software engineering–based Agile Methodologies under SCRUM principles (empiric control of the development process using continuous feedback procedures). The whole mobile app functionality and its user interfaces and interactions will be designed under the premise of maximizing the user experience in order to boost adherence to treatment.

All of the communications between the mobile app and the supporting backend will be protected by means of an SSL digital certificate on the server, which will provide end-to-end encrypted communications.

#### Software: Control of Adherence to the App

The software used in the study is based on a technology that reports the access and time that a person been in the app. The app also has a reminders and warnings that will guarantee that patients can use it and not discontinue treatment.

### Intervention Programme

Before designing the mobile app, a focus group with young people with psychosis was carried out at the Child and Adolescent Psychiatry Department, Gregorio Marañon Hospital. The opinions and contributions of the participants on the design of the modules were very useful for the design of the mobile app.

#### Mobile Phone Psychotherapy App

The psychotherapy app programme is composed of five modules:

-Psychoeducational module
-Module on recognition of symptoms and prevention of relapses
-Problem-solving module
-Mindfulness module
-Contact wall module

The modules will be assessed in parallel throughout the study. Each module is described in detail below.

##### Psychoeducational Module

The psychoeducational module focuses on improving patients' insight into their illness, adherence, detection and identification of prodromes, early intervention to prevent relapses, healthy lifestyles and techniques for managing anxiety, social skills, and consumption of toxic substances. In order to facilitate integration, psychoeducation will be administered through animated videos (comic-like format) of about 2–3 min in duration, which were made and edited by clinical experts in psychosis.

The 12 sessions are included in module are in Table 1.

**Table 1:** Online sessions of the psychoeducation module.

**Number of session**	**Title**
1.	What is a first episode of psychosis? Protective and risk factors
2.	Recognition of symptoms (positive, negative, and affective)
3.	Treatment (types and adherence)
4.	Treatment (adverse effects)
5.	Drugs and psychosis
6.	Prevention of relapses: Protective and risk factors
7.	How to act in a crisis
8.	Healthy lifestyle I: Stress
9.	Healthy lifestyle II: Protective factors in psychosis
10.	Social skills: Communication and assertiveness techniques
11.	Adolescence
12.	New technologies

All of the selected sessions are based on previous studies of that have shown the efficacy of psychoeducational interventions in adolescents with FEP (61–64).

The program will automatically check whether the video has been watched. In addition, it will be accompanied by a 5-item questionnaire about each video to guarantee that the patient has understood the content.

##### Module on Recognition of Symptoms and Prevention of Relapses: Alert System

This module will add some important novelties. It has an alert system by which patients will be able to check their symptoms, cognitive performance, and emotional and behavioral circumstances *weekly*. The system will return feedback with specific recommendations. This module is based on previous studies that have shown efficacy for FEP patients (20, 21). For example, if a patient begins to spend more time at home, becomes more isolated, and complains of having less energy, the system, automatically, will send recommendations to the patient via the mobile app. Some of the recommendations could be as follows: “We feel that you are changing your habits, it seems you are more inactive. We recommend you to try to go for a walk, even around the area of your house; to try not to get into bed and try to do some activities that you liked before. If you need to talk to a friend or family member to encourage you and accompany you to prevent the situation from becoming worse, please do so. Nevertheless, we recommend you to talk with your psychiatrist or psychologist to see what is happening.” This system, for example, also gives recommendations for visiting the psychiatrist or psychologist or for hygiene activities.

##### Problem-Solving Module

> In this module, the participants can share their problems and ask other participants to provide possible solutions. All participants have access to a problem-solving wall. The module is based on McFarlane's Multiple Family therapy model and on the application of problem-solving techniques that have proven efficacious for patients with severe mental disorders and their relatives (8, 61–64). A moderator (psychologist or psychiatrist specialized in FEP) will review the contents of the forum and lead participants to relevant topics if necessary.

##### Mindfulness Module

> In this module, we will explain the concept of mindfulness and its objectives with a 3-min video. The module consists of 3 recordings made by clinicians who are experts in mindfulness, in which three techniques of this type of therapy are developed. The module will be taught by means of audio recordings in which the techniques described will be body scan and attention focusing (breathing and breathing) to feel better (self-compassion). The aim of the module is to help to reduce anxiety and to increase the sensation of control. This type of intervention has demonstrated efficacy with psychosis (21, 65), even when administered via mobile technology (66).

##### Contact Wall Module

The contact wall module will include a contact wall based on previous studies that have shown efficacy in FEP (20). Moreover, a review about this strategy concluded that a contact wall can improve social contact and reduce isolation in patients with psychosis (67). Patients can use the wall to share their interests and experiences. The module can help to develop sociability and will bring a sense of normality, favoring activities according to age and preferences. It does not necessarily focus on the disease. The contact wall aims to act as a social space, within the app, where patients can exchange information on their leisure interests. Information published on the contact wall is supervised and previously approved by a therapist, who acts as a moderator.

#### Treatment As Usual (TAU)

Treatment as usual refers to the standard treatment provided in the Spanish National Health Service. For patients with FEP, this consists of ambulatory psychopharmacotherapy and regular sessions with an assigned psychiatrist. In some cases, it involves outpatient treatment with a clinical psychologist, thus enabling patients to manage their condition themselves.

## Anticipant Results

The study anticipates that adolescent patients with psychosis of the experimental group, after the application of the mobile app intervention, will improve, significantly, their symptoms, adherence, awareness, prognosis and quality of life and will reduce the drugs consumption, compared with the control group. The study hypothesis is that the online treatment will be more effective than the treatment as usual intervention in reducing the indicated variables.

## Statistical Analysis

Data will be analyzed on an intention-to-treat (ITT) basis (68). In accordance with the ITT principle, all patients will be included in the analysis, independently of the groups to which they are randomly assigned and regardless of whether they complete the treatment or not.

Outcomes will be reported with 95% confidence intervals. All data will be analyzed using SPSS version 21.0. The level of bilateral significance for all statistical tests will be established at α = 0.05.

The baseline characteristics of the sample will be compared between the two groups using the Pearson chi square test for the categorical variables and the *t* test or Mann-Whitney test for the quantitative variables.

Mixed model repeated-measures analysis will be used to explore the following effects: group × time interaction between the control group and the intervention group for clinical and functional variables during the follow-up period. Intergroup and intragroup differences during follow-up will also be studied.

The effect sizes will be calculated to quantify the effect of the intervention on the groups. To consider a certain value a large or small effect size, the following orientations will be accepted: *d* = 0.20 (small), *d* = 0.50 (moderate), and *d* = 0.80 (large) (69).

The research results will be reported in agreement with the Consolidated Standards of Reporting Trials (CONSORT) 2010 statements (70, 71) and the Standard Protocol Item: Recommendations for Interventional Trials (SPIRIT) guidelines (72, 73).

## Dissemination

The final database will be the property of the research team and shall not be shared without the principal investigator's permission. The dissemination plans include presentations at local, national, and international scientific conferences.

## Discussion

We describe an innovative online intervention which will be administered through a mobile application specifically designed for adolescents with FEP. Previous studies have demonstrated the acceptance and positive perception of this type of intervention in FEP patients and the absence of associated clinical and safety problems. In addition, preliminary results on efficacy with regards to symptoms (psychotic: positive and negative symptoms and affective symptoms), adherence, sociability, and admissions are favorable (20, 21, 23). The intervention is appropriate for patients with psychosis, especially young people, because it does not require patients to modify their daily activities, such as going to high school or meeting with friends. Accordingly, some authors have pointed out that this type of intervention could create opportunities to overcome the limitations of face-to-face treatment (economic, difficulties in accessing health services, etc.) (15, 23, 74–78) and thus may imply major benefits for the health system and society in general.

Psychotherapy interventions based on a mobile app (psychoeducation, problem solving model, and mindfulness model) have proven to be effective in psychosis, mostly in a face-to-face format (8, 9, 79–82), and this modality might facilitate this type of intervention.

The results of this study could complement traditional treatment, especially for young people with psychosis in the critical period of their disease. Consequently, applying the intervention at the onset of the disorder will enable the patients to achieve the benefits of early treatment, not only at the pharmacological level, but also in the areas addressed by adjunctive treatments that have proven to be effective (83).

Our study is subject to a series of limitations. First, the number of patients receiving treatment is limited, and the follow-up is short. However, a previous study about online interventions (84) in patients with recent-onset schizophrenia spectrum disorders reported good results with the same length of follow-up and a similar sample size. Second, some of the variables collected will be measured using self-reported instruments by the app (with the corresponding risk of under- or overestimation of symptoms), although patients will also be assessed by clinicians and the scales applied will be among the most widely used by clinicians and researchers in this type of patients. Nevertheless, research suggests that symptom indices obtained from individuals with psychosis through smartphone technology have a higher concordance with the clinician's scores than “paper and pencil” self-assessments (85). Third, participants must have a smartphone to receive the psychological intervention; however, research suggests that up to 90% of people with FEP have access to a smartphone (16, 17). Four, there may be low adoption and retention of mobile app interventions in samples of patients with severe mentally illness. However, a large number of studies and reviews have reported good results in this sense (between 71 and 93%) (86) and shown that the simplicity of access and the attractiveness and content of the online treatment improve these aspects (19). Our mobile app intervention brings these user-friendly characteristics together. In addition, the system sends reminders and warnings to the patients to ensure that patient adhere to treatment.

This is a pilot study, an application with a larger number of patients and a longer follow-up period may have a greater impact. However, very few methodologically rigorous studies are currently assessing this mobile app–based therapy. Therefore, it is necessary to develop studies that use a valid and randomized methodology (23, 87).

## Conclusion

Ours is an innovative pilot study that assesses a psychological intervention through a mobile app for patients with FEP during the critical period. The study could make treatment available to a much larger population and with more difficult access to face to face therapy.

To our knowledge, this is the first randomized online treatment for adolescent with early psychosis comparison with treatment as usual. The main novelty of our approach is the integration of new technologies with traditional psychoeducation, cognitive behavioral therapy techniques, mindfulness and social skills development.

## Ethics Statement

The Ethics Committees of the University International of La Rioja and Hospital General Universitario Gregrorio Marañón (March 21, 2017, Certificate no. 06/2017) approved this study. All patients will be provided with written and oral information about the study, prior to giving their consent to participate.

This study is registered with the international standard randomized controlled trial number NCT (www.clinicaltrials.gov).

## Author Contributions

SB and AC wrote the first draft of the manuscript. All of the authors participated in the drafting of the manuscript and all of them approved the final version. TS-G, MaM, SB, and AC participated in the clinical design of the online intervention and the design of the clinical assessment protocol. SR-A and AC participated in the technical design of the online intervention. AC is the principal investigator and participated in the study design, writing, editing, and all of the components of the project.

### Conflict of Interest Statement

The authors declare that the research was conducted in the absence of any commercial or financial relationships that could be construed as a potential conflict of interest.
